# The Art of Supervision: Perspectives from Psychiatric Trainees on Effective Supervision

**DOI:** 10.1192/bjo.2026.11340

**Published:** 2026-06-30

**Authors:** Rhian Bradley

**Affiliations:** Kent and Medway Mental Health NHS Trust, Dartford, United Kingdom

## Abstract

**Aims:**

Educational, clinical and psychiatric supervision play a crucial role in the development of psychiatric trainees, contribute to safe clinical practice, and promote trainee wellbeing. Whilst resident doctors express high levels of satisfaction with supervision within the National Training Survey, the lived experience of psychiatric trainees of supervision is not well understood; evidenced within a systematic review of the literature. Thus, the aim of this project was to explore the experience of psychiatric trainees in Kent and Medway Mental Health NHS Trust (KMMH) of supervision, with a focus on key factors affecting their satisfaction.

**Methods:**

A focus group, qualitative methodology was used. Fifteen core and higher psychiatric trainees were recruited through purposive sampling. Four focus groups were conducted via Microsoft Teams. Anonymised transcripts were verified through member checking. Data saturation was achieved. Ethical approval was granted from the University of Kent.

**Results:**

Participants described beneficial aspects of supervision and areas that were more challenging. Data was analysed using reflexive thematic analysis. Five themes emerged:

Scheduled Regular Supervision: Not only being able to access regular supervision, but for this to be scheduled, enabling trainees to plan the agenda in advance.

Knowledge of Supervision: For supervisors and trainees to understand the value of a shared agenda and the remit of supervision, specifically the distinction between clinical and educational supervision. Participants described difficulties when supervision was trivialised through supervisors equating brief informal discussions with required weekly reflective one-to-one supervision.

Support Across the Training Journey: Adapting the supervisory offer in response to differing levels of experience and key transition points was perceived as helpful, particularly for international medical graduates.

Fostering Mutual Respect: Enablers of a mutually respectful relationship were flattening the traditional power hierarchy and engaging in bi-directional feedback; barriers to providing feedback were described.

Psychological Safety: Adopting a non-judgemental stance towards trainees was recognised as important, alongside supervisors making genuine efforts to ‘check in’, avoiding comparing trainees to others, and conducting supervision in a private space.

**Conclusion:**

The project has contributed to a deeper understanding regarding psychiatric trainees’ experience of supervision, not only factors perceived as helpful but also the complexity and nuance required; captured adeptly by a trainee “*that's why there's such an art to supervision*”. Recommendations are proposed: To foster conditions that promote helpful supervisory behaviours; to empower trainees within their supervision; and co-create feedback systems that recognise and respond to unhelpful supervisory behaviours without fear of adverse repercussions.

